# Automated quality control for a molecular surveillance system

**DOI:** 10.1186/s12859-018-2329-5

**Published:** 2018-10-22

**Authors:** Seth Sims, Atkinson G. Longmire, David S. Campo, Sumathi Ramachandran, Magdalena Medrzycki, Lilia Ganova-Raeva, Yulin Lin, Amanda Sue, Hong Thai, Alexander Zelikovsky, Yury Khudyakov

**Affiliations:** 1 Division of Viral Hepatitis, Centers for Disease Control and Prevention, 1600 Clifton Road, MS A33, Atlanta, GA 30329 USA; 2 Department of Computer Science, Georgia State University, 1 Park Place, room 623, Atlanta, GA 30302 USA; 3 Northrop Grumman Corporation, 2800 Century Pkwy NE, Suite 600, Atlanta, GA 30345 USA

**Keywords:** HVR1, HCV, Transmission, Outbreak detection, Molecular surveillance, Quality control

## Abstract

**Background:**

Molecular surveillance and outbreak investigation are important for elimination of hepatitis C virus (HCV) infection in the United States. A web-based system, Global Hepatitis Outbreak and Surveillance Technology (GHOST), has been developed using Illumina MiSeq-based amplicon sequence data derived from the HCV E1/E2-junction genomic region to enable public health institutions to conduct cost-effective and accurate molecular surveillance, outbreak detection and strain characterization. However, as there are many factors that could impact input data quality to which the GHOST system is not completely immune, accuracy of epidemiological inferences generated by GHOST may be affected. Here, we analyze the data submitted to the GHOST system during its pilot phase to assess the nature of the data and to identify common quality concerns that can be detected and corrected automatically.

**Results:**

The GHOST quality control filters were individually examined, and quality failure rates were measured for all samples, including negative controls. New filters were developed and introduced to detect primer dimers, loss of specimen-specific product, or short products. The genotyping tool was adjusted to improve the accuracy of subtype calls. The identification of “chordless” cycles in a transmission network from data generated with known laboratory-based quality concerns allowed for further improvement of transmission detection by GHOST in surveillance settings. Parameters derived to detect actionable common quality control anomalies were incorporated into the automatic quality control module that rejects data depending on the magnitude of a quality problem, and warns and guides users in performing correctional actions. The guiding responses generated by the system are tailored to the GHOST laboratory protocol.

**Conclusions:**

Several new quality control problems were identified in MiSeq data submitted to GHOST and used to improve protection of the system from erroneous data and users from erroneous inferences. The GHOST system was upgraded to include identification of causes of erroneous data and recommendation of corrective actions to laboratory users.

## Background

Epidemiological surveillance has been a cornerstone of all disease elimination programs [1–7] to measure incidence, prevalence, and effectiveness of intervention. Molecular surveillance is the collection of genomic information, from which usable public health information can be inferred, and constitutes a powerful complement to traditional epidemiological surveillance [8]. In outbreak settings, molecular surveillance provides key information for initial identification, source attribution, and accurate identification of the cases associated with a disease cluster.

Due to the recent development of effective treatment options [9, 10], an elimination strategy for the hepatitis C virus (HCV) has been developed in the United States [7] and worldwide [11]. Molecular surveillance is recommended by the National Academies of Science, Engineering, and Medicine as a key tool in addressing the dynamics and historical reconstruction of transmission that will inevitably vary by population, location, and behaviors [7].

The Global Hepatitis Outbreak and Surveillance Technology (GHOST) is a system that integrates amplicon-based next-generation sequencing, bioinformatics and information technologies for molecular surveillance and outbreak investigation. It accepts Illumina MiSeq-sequence data, and produces a network graph showing which cases are linked by a common viral strain [12]. The GHOST distance method for transmission detection was developed on End-Point Limited Dilution (EPLD) data and validated on the 454 sequencing technology [13]. A number of computational approaches were explored for scaling this method to larger datasets [14], and some of these approaches were implemented into the GHOST system as it was adapted to the Illumina MiSeq sequencing technology [12].

Owing to significant complexity of sequence data gathering and interpretation requiring specialized molecular epidemiological and bioinformatics expertise, molecular surveillance of infectious diseases is mainly a subject of academic research and practiced generally by most technically advanced institutions, which hinder a broad application of molecular surveillance to public health interventions. GHOST is designed to reduce this complexity and to enable users to conduct efficient and accurate molecular surveillance and outbreak investigation irrespective of the users’ level of expertise. An overriding goal throughout system development was simplicity of use and interpretation. The nature of bloodborne transmission events, as well as distance methods used to detect them are quite well suited to a simple and intuitive network graph. However, behind the simplistic interface is a complex computational and mathematical system that can be influenced by irregular input and induced to produce misleading results. Thus, control of quality of the data and information generated is fundamentally important for the system’s practical application in public health.

Here, a common set of problem sources was identified by observing submissions made to GHOST, solutions to fielded support requests, and statistical examination of the data. Analyses were used to improve and upgrade the quality assurance module in GHOST to provide a clear path of corrective action for GHOST users when laboratory issues are encountered, and in so doing, bridge the bioinformatics gap between laboratory sequencing production and actionable epidemiological information.

## Methods

The data used in this quality control study were submitted by participating public health institutions in the GHOST projects initial pilot phases between 11/27/2016 and 8/22/2017 and contained sequences from 3181 samples originating from 173 GHOST quality control (QC) tasks. Samples were deduplicated by computing the CRC32 hashing algorithm for all sample files and discarding samples for which the CRC32 values were already found. The GHOST sequencing protocol specifies the inclusion of at least one negative control sample in each library sequenced. Negative control samples were discriminated from the positives (non-negative controls) using a pre-defined set of strings commonly observed to search sample names.

Using a high performance distributed cluster, all samples were re-executed for standardization with the most recent GHOST version. The version included a new filter to measure the existence of primer dimers or non-specifc product. For each read pair, this filter inspects the forward read for the forward primer sequence using the same search parameters as the filter dedicated to primer verification and read orientation. Once found, the reverse complement is searched for the reverse primer sequence. If the distance between the forward and reverse primer sequences is found to be less than the threshold set in the filter dedicated to read length (185 bp), the pair is discarded. If both primer sequences are not found, the process is repeated with the reverse read. Descriptions of all other filters are detailed in Longmire et al., 2017 and briefly described in Table 1. The GHOST command line switch “failed_precious” was used to signal continued processing of samples that would normally cease processing if falling below any predefined threshold set by default on some filters.

**Table 1:** GHOST QC filters listed in order of execution. All filters except for “Primer dimer” are discussed in detail in Longmire et al., [12]

Order	Filter name	Description	Position relative to *N* = 20,000 random sampling
1	Ambiguity	After standard demultiplexing, read pairs are filtered out if a read has more than three N’s.	Before
2	Primer dimer	Checks for the existence of primer dimers or non-specifc product. For each read pair, this filter inspects the forward read for forward primer using the same search parameters as the filter dedicated to primer verification and read orientation. Once found, the reverse complement is searched for the reverse primer. If the distance between the forward and reverse primers is found to be less than the threshold set in the filter dedicated to read length (185 bp), the pair is discarded. If both primers are not found, the process is repeated with the reverse read.	Before
3	Short read	Read pairs are filtered out if either read has a length less than 185 bp.	Before
4	MID mismatch	Each identifier on both forward and reverse reads are examined and the pair is discarded if either identifier is found to not be an exact match to a given list of valid identifiers.	Before
5	Minority MID	Pairs containing valid identifiers are discarded if they are not a constituent of the majority identifier tuple. If 25% or more of the read pairs are found to contain valid identifiers that are not the majority tuple, the entire sample is discarded from analysis without further processing.	Before
6	Primer verification	Primer sequence patterns are searched for in the forward and reverse reads. Primer sequences are located in each read using fuzzy matching and only allow substitutions ≤2, insertions (relative to the reference) ≤ 1, deletions (relative to the reference) ≤ 1, and a combination of total errors ≤3. Read pairs where either primers cannot be found are discarded. The primer locations are used to orient the reads into the uniform orientation.	After
7	Casper mismatch	Read pairs are unified into a single error-corrected sequence using the Casper error correction method with a quality threshold of 15, k-mer length of 17, k-mer neighborhood of 8, and minimum match threshold of 95%. Overlap fitness is evaluated by the classical Hamming Distance. The overlap corresponding to the highest ratio of correct positions to overlap length is selected, with the longest overlap being preferred in the event of there being more than one overlap with equal ratios.	After
8	Nonsense	Merged sequences are discarded if a nonsense-free reading frame cannot be found.	After

**Fig. 1:**
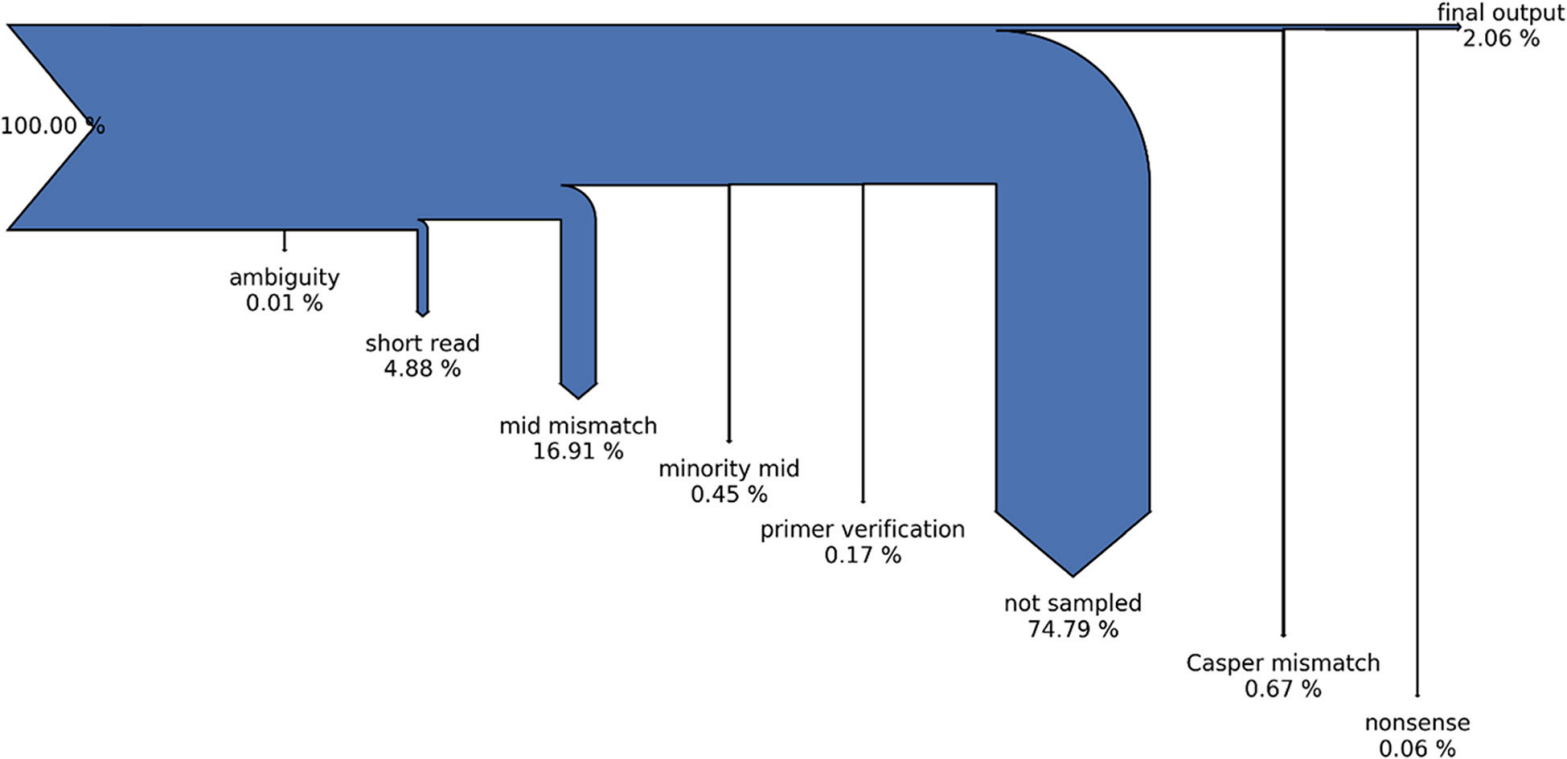
Sankey diagram of read pair allocation for all samples after deduplication. Arrow thickness represents the proportion of read pairs removed by the filter step. The “not sampled” step represents those reads not used after 20,000 read pair random sampling

The GHOST QC task has two phases of filtering. The first phase operates on all read pairs in a sample. The second phase only operates on a subset of 20,000 read pairs randomly selected from those that passed all filters in the first phase. In both phases, each filter operates in succession using only read pairs passing the previous filter, or the number of reads selected in the random sample which is 20,000 (Fig. 1, Table 1) [12].With these filter phases in mind, a Python script was created to normalize each filter result to a percentage with respect to the number of reads that entered the filter. Welch’s *t*-test for difference of means with unequal variance was used to calculate the difference in filter result means between sample groups using the Holm-Sidak method to control for multiple testing as implemented in the Python package Statsmodels [15].

The GHOST Laboratory Standard Operating Procedure includes multiple intentionally negative samples as controls. We use the characteristics of these samples to produce a classifier to recognize negative samples. Users occasionally introduce unintentional negative samples through mislabeling or loss of amplification product. Deduplicated samples were partitioned into 4 categories: Passing Non-Negative (PNN), Failing Non-Negative (FNN), Passing Negative (PN), and Failing Negative (FN) and evaluated for Quality Control task filtering performance (Table 2). The 3 filters found to be most significant to detection of negative characteristics were used to employ an exhaustive grid search from 0 to 100% for each variable with 0.5% increments. The Gini impurity index of the PNN set in comparison to the combined FN and PN sets was evaluated for each variable combination, and the index with the minimum index was determined to be the best fit.

**Table 2:** Deduplicated data partitioned into 4 categories

Failing non-negative	225
Passing non-negative	1750
Failing negative	87
Passing negative	25
Total	2087

The current GHOST subtyping classification uses blast algorithms to query a predetermined curated reference set, and the best hit is selected. If the best match is of poor quality (bit score-derived log probability > − 135), it is disregarded and labeled with the “unmatched” subtype along with sequences with no match [12]. Subtype classification for all deduplicated samples was analyzed to determine the distance between the first and second-best hits. For those with 2 or more matches, the ratio of best to the second-best hit was measured to determine the precision of the call using the bit score-derived log probabilities as the hit values.

Logistic regression analysis was applied multiplexing level and flowcell type to determine any statistical relationship to QC task passage. Finally, all deduplicated data from the collection time period were analyzed together for linkage to determine if any unexpected links exist suggesting intra- or inter-run contamination. Due to the computational load and durational requirements, the linkage analysis was broken into four separate tasks, and a python script was used to compile the outputs into a single unified result. The Gephi v0.9.1 software [16] was used to visualize linkage from all sample data sets. This study is an effort to control quality though system-level parameter optimization and protocol-specific feedback. No personally identifiable information (PII) is contained herein.

## Results

### Overview of production data

The 173 GHOST submissions containing sequence data from 3181 samples were analyzed. Deduplication reduced the sequence data to 2087 originating samples. 312 (14.95%) failed the GHOST QC task, and 112 (5.37%) were identified as negative control samples; 22.32% (25/112) of negative control samples passed the QC task.

### Primer-dimer filter performance

**Fig. 2:**
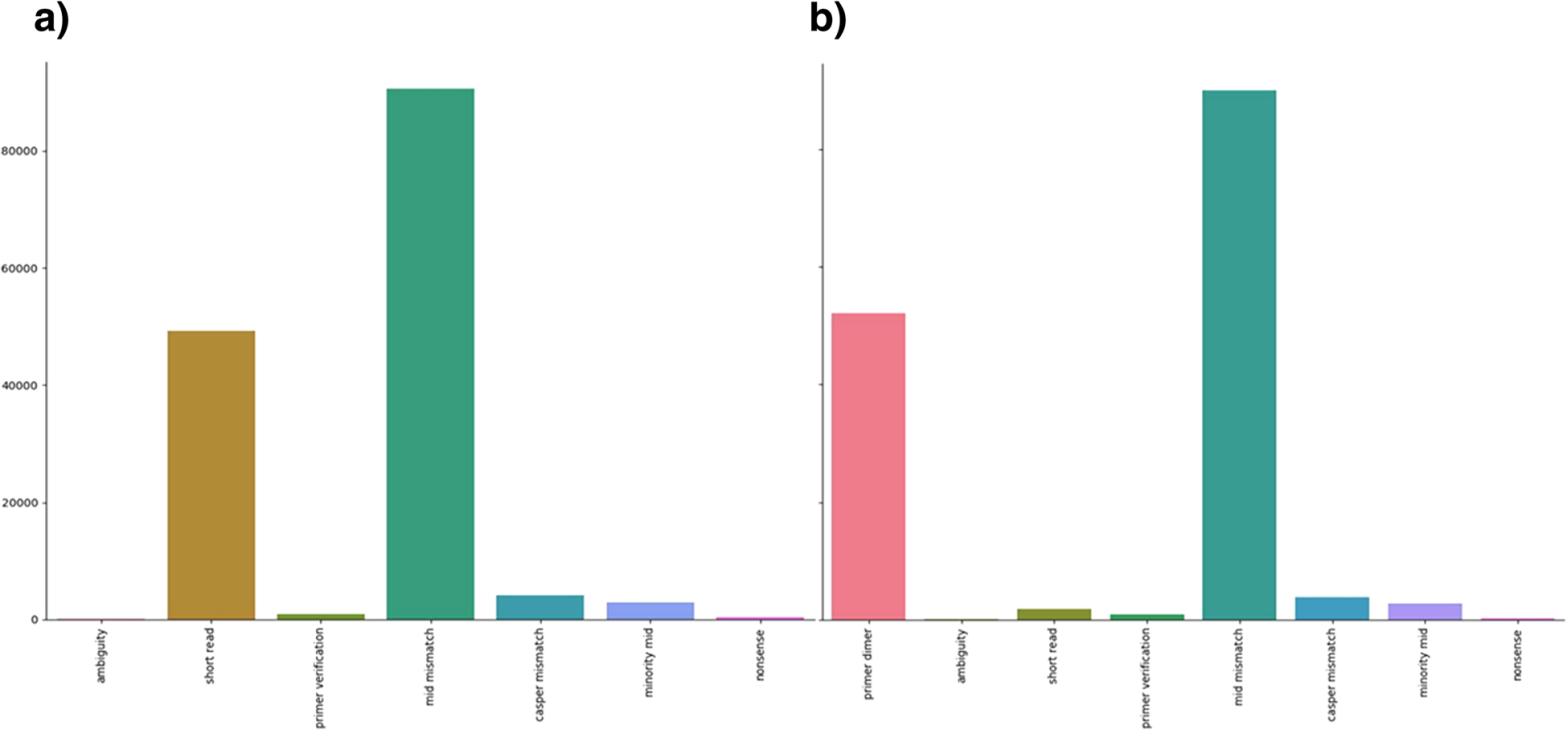
Performance of primer dimer filter. **a**) Mean values of filters before the introduction of the primer dimer filter. **b**) Mean value of filters after introduction of the primer dimer filter

**Fig. 3:**
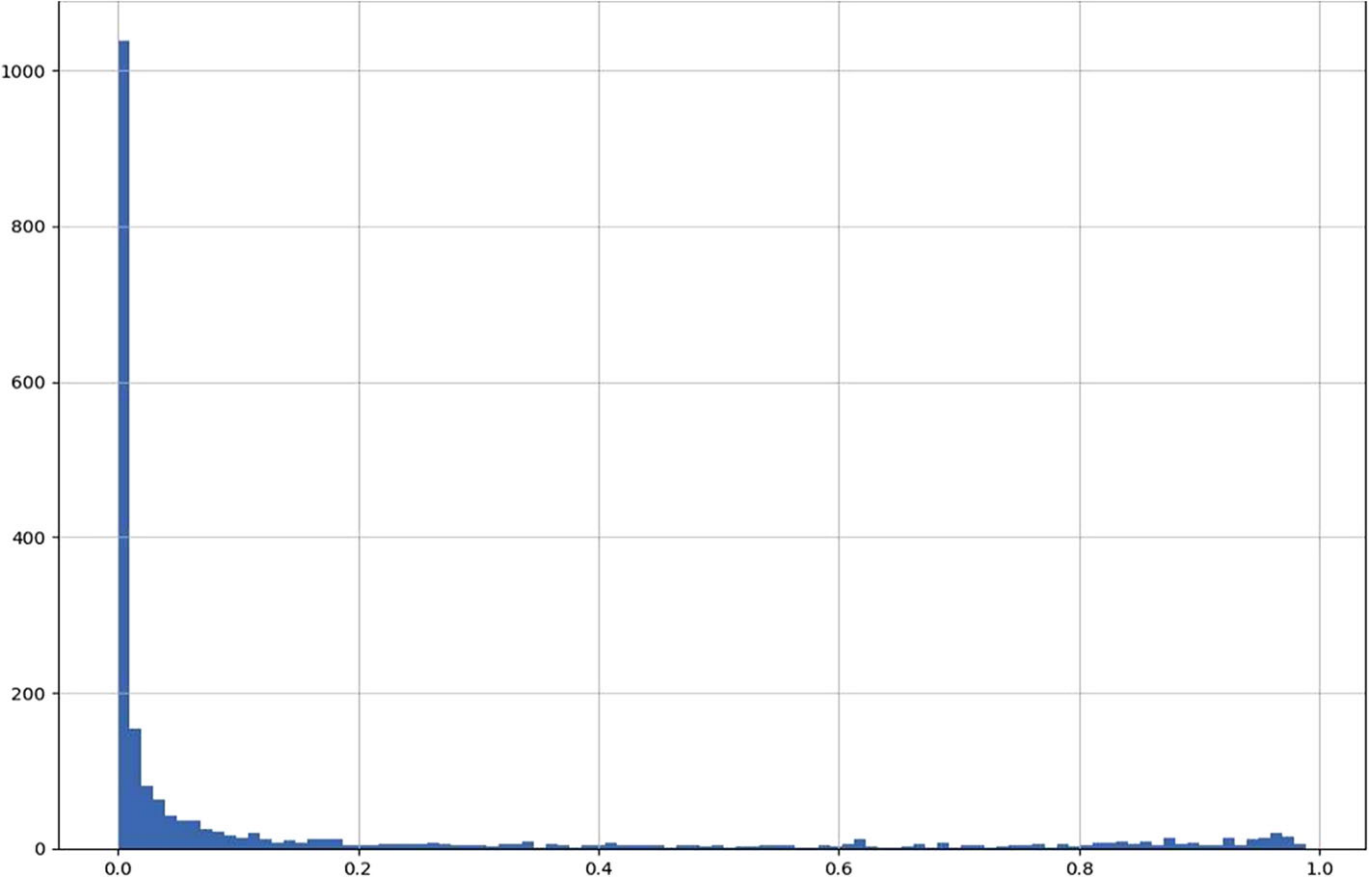
Histogram of primer dimer filter normalized values. Normalization is calculated with respect to the number of read pairs entering into the filter

**Fig. 4:**
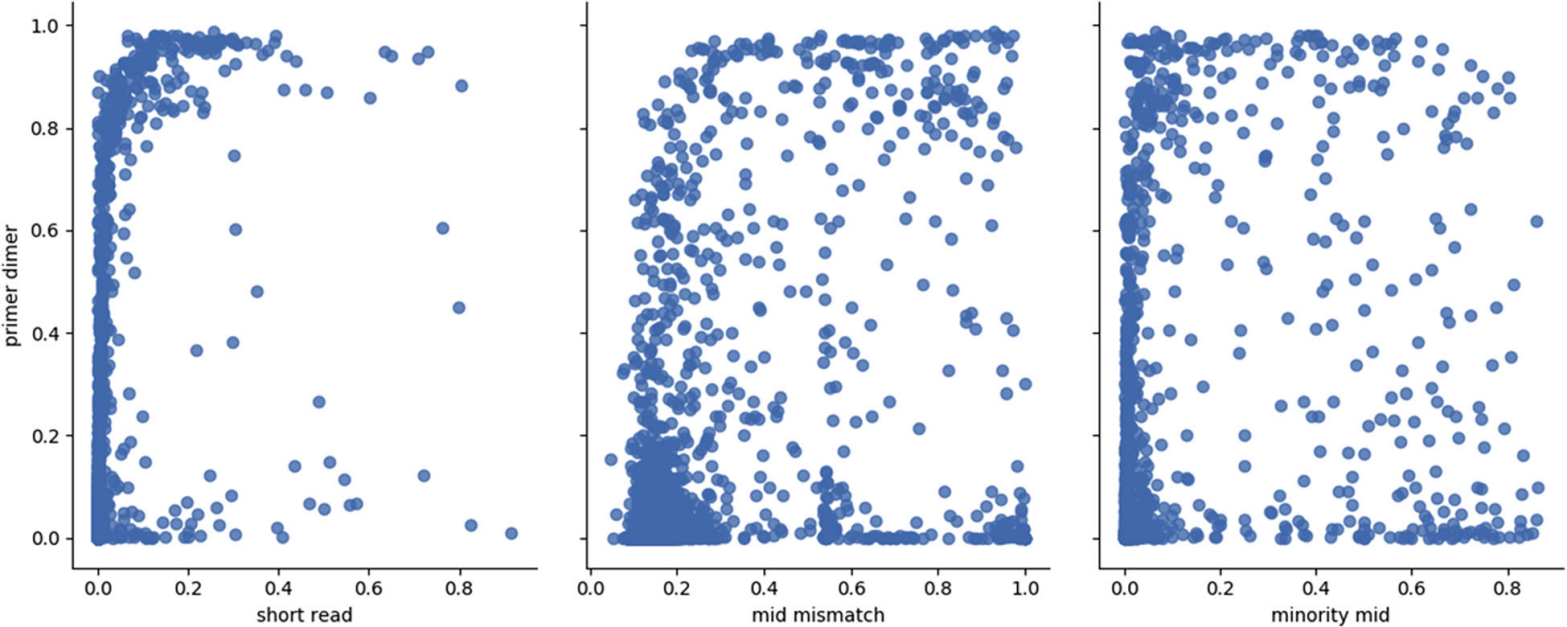
Scatter plots of the primer dimer filter compared against 3 other filters

Comparison of mean filter results for all samples with that of mean filter levels before the primer dimer filter was implemented revealed that most read pairs previously being discarded by the read length filter were absorbed by the primer-dimer filter (Fig. 2). Distribution of discarded pair sample percentages for the primer-dimer filter were concentrated at levels close to zero, however, sample percentages were evenly dispersed at low level throughout the spectrum (Fig. 3). It was observed that at ~ 80% and above, the primer-dimer filter rate is associated with elevation in the rate of pairs discarded by the read length, MID mismatch, and minority MID filters (Fig. 4).

### Filter performance

**Fig. 5:**
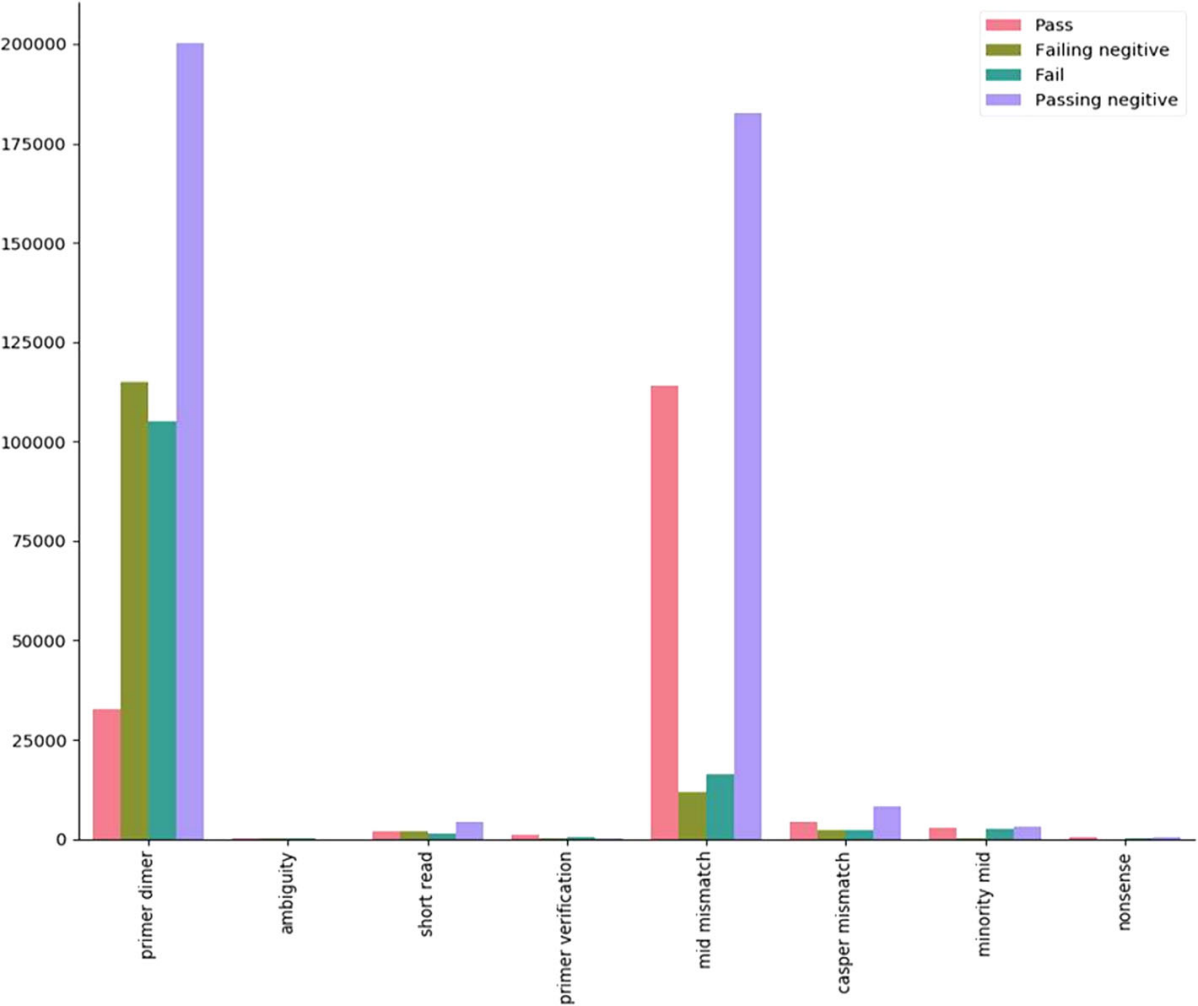
Mean values (before normalization) of all GHOST QC task filters with respect to the 4 mutually exclusive sample sets

**Fig. 6:**
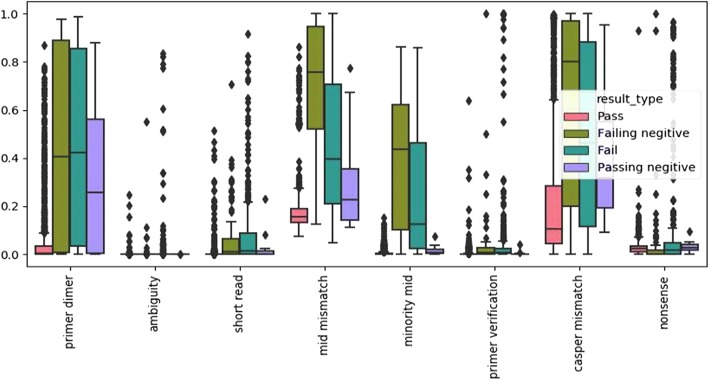
Boxplots of filter distributions after normalization for all deduplicated samples

The primer dimer and MID mismatch filters displayed significantly higher means of PN over PNN (Fig. 5). After values were normalized with respect to the number of read pairs entering the filter, the primer dimer, MID mismatch, and Casper mismatch filters all show significant elevation in PN with respect to PNN (Fig. 6).

Welch’s t-test on filter results and sequence statistics between the PNN and FNN sets showed no statistically significant evidence for rejection of the null hypothesis. Comparison between PN and PNN showed statistically significant differences in means for maximum read length, and all filters, particularly the primer dimer, best MID mismatch, and minority MID filters with *p*-values of 2.919E-67, 1.447E-66, and 1.667E-58 respectively (Table 3). When applied to the PN and FN, only the minority MID and best MID mismatch filters showed statistical significance with p-values of 1.590E-24 and 1.447E-4 respectively (Table 3).

**Table 3:** Welch’s test for mean comparison of means

Comparison	Filter	*t*	*p*-value	Corrected *p*-value	Reject
P vs F	Primer dimer	2.485304	3.220E-02	7.210E-01	FALSE
P vs F	Ambiguity	−2.64516	8.258E-03	2.999E-01	FALSE
P vs F	Short read	1.227051	2.478E-01	9.995E-01	FALSE
P vs F	MID mismatch	1.721249	1.159E-01	9.828E-01	FALSE
P vs F	Minority MID	1.491232	1.666E-01	9.949E-01	FALSE
P vs F	Primer verification	0.318833	7.561E-01	1.000E + 00	FALSE
P vs F	Casper mismatch	2.09018	6.284E-02	9.033E-01	FALSE
P vs F	Nonsense	0.600825	5.611E-01	1.000E + 00	FALSE
P vs F	raw pairs passed	−1.65813	1.279E-01	9.875E-01	FALSE
P vs F	r1_maxlength	0.027582	9.785E-01	1.000E + 00	FALSE
P vs F	r2_maxlength	0.027582	9.785E-01	1.000E + 00	FALSE
P vs F	r1_numseqs	0.496099	6.303E-01	1.000E + 00	FALSE
P vs F	r2_numseqs	0.496099	6.303E-01	1.000E + 00	FALSE
P vs F	r1_minlength	−2.61235	2.243E-02	6.055E-01	FALSE
P vs F	r2_minlength	−1.40858	1.889E-01	9.972E-01	FALSE
P vs F	r1_gc	0.9154	3.810E-01	1.000E + 00	FALSE
P vs F	r2_gc	1.115745	2.897E-01	9.999E-01	FALSE
P vs F	r1_qual	−0.7403	4.759E-01	1.000E + 00	FALSE
P vs F	r2_qual	−0.22578	8.259E-01	1.000E + 00	FALSE
PN vs PNN	Primer dimer	−20.708	2.919E-67	0.000E + 00	TRUE
PN vs PNN	Ambiguity	−3.81125	1.589E-04	7.125E-03	TRUE
PN vs PNN	Short read	−9.98183	3.000E-21	0.000E + 00	TRUE
PN vs PNN	MID mismatch	−20.5454	1.447E-66	0.000E + 00	TRUE
PN vs PNN	Minority MID	−18.9948	1.667E-58	0.000E + 00	TRUE
PN vs PNN	Primer verification	−6.14462	1.853E-09	9.082E-08	TRUE
PN vs PNN	Casper mismatch	−14.2144	3.668E-39	0.000E + 00	TRUE
PN vs PNN	Nonsense	−6.44656	3.119E-10	1.559E-08	TRUE
PN vs PNN	raw pairs passed	15.95968	1.747E-47	0.000E + 00	TRUE
PN vs PNN	r1_maxlength	3.883107	1.138E-04	5.446E-03	TRUE
PN vs PNN	r2_maxlength	3.883107	1.138E-04	5.446E-03	TRUE
PN vs PNN	r1_numseqs	2.021773	4.358E-02	8.161E-01	FALSE
PN vs PNN	r2_numseqs	2.021773	4.358E-02	8.161E-01	FALSE
PN vs PNN	r1_minlength	0.927906	3.538E-01	1.000E + 00	FALSE
PN vs PNN	r2_minlength	−0.5458	5.854E-01	1.000E + 00	FALSE
PN vs PNN	r1_gc	−0.26695	7.896E-01	1.000E + 00	FALSE
PN vs PNN	r2_gc	−0.90928	3.635E-01	1.000E + 00	FALSE
PN vs PNN	r1_qual	0.559492	5.760E-01	1.000E + 00	FALSE
PN vs PNN	r2_qual	3.245425	1.234E-03	5.290E-02	FALSE
PN vs FN	Primer dimer	−1.03201	3.207E-01	9.999E-01	FALSE
PN vs FN	Ambiguity	−1.51352	1.333E-01	9.881E-01	FALSE
PN vs FN	Short read	−1.47375	1.586E-01	9.944E-01	FALSE
PN vs FN	MID mismatch	−5.29945	1.447E-04	6.634E-03	TRUE
PN vs FN	Minority MID	−13.2917	1.590E-24	0.000E + 00	TRUE
PN vs FN	Primer verification	−2.51412	1.338E-02	4.321E-01	FALSE
PN vs FN	Casper mismatch	−1.91524	7.643E-02	9.381E-01	FALSE
PN vs FN	Nonsense	−0.52093	6.037E-01	1.000E + 00	FALSE
PN vs FN	raw pairs passed	2.537808	2.448E-02	6.290E-01	FALSE
PN vs FN	r1_maxlength	0.388275	7.045E-01	1.000E + 00	FALSE
PN vs FN	r2_maxlength	0.388275	7.045E-01	1.000E + 00	FALSE
PN vs FN	r1_numseqs	0.79751	4.400E-01	1.000E + 00	FALSE
PN vs FN	r2_numseqs	0.79751	4.400E-01	1.000E + 00	FALSE
PN vs FN	r1_minlength	−1.71001	9.675E-02	9.686E-01	FALSE
PN vs FN	r2_minlength	−0.98698	3.430E-01	1.000E + 00	FALSE
PN vs FN	r1_gc	0.433694	6.711E-01	1.000E + 00	FALSE
PN vs FN	r2_gc	0.353036	7.286E-01	1.000E + 00	FALSE
PN vs FN	r1_qual	−0.67196	5.144E-01	1.000E + 00	FALSE
PN vs FN	r2_qual	0.15547	8.790E-01	1.000E + 00	FALSE

*P pass, F fail, PN passing negative, PNN passing non-negative, FN failing negative*

### Discrimination of negatives

**Fig. 7:**
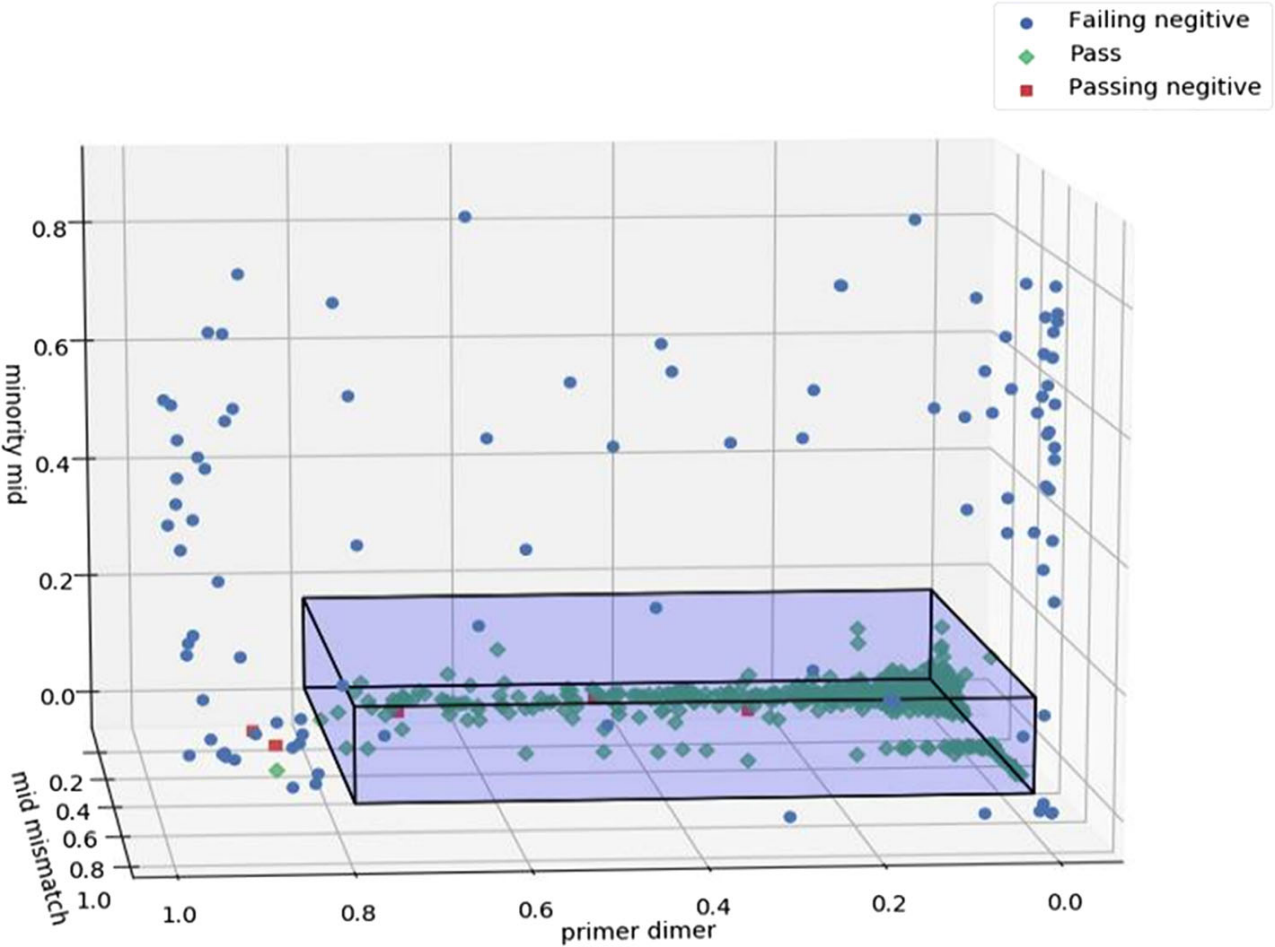
Scatter plot showing samples in categories PNN, FN, and PN. Box shows the application of the three threshold combination using minimization of Gini impurity index

**Fig. 8:**
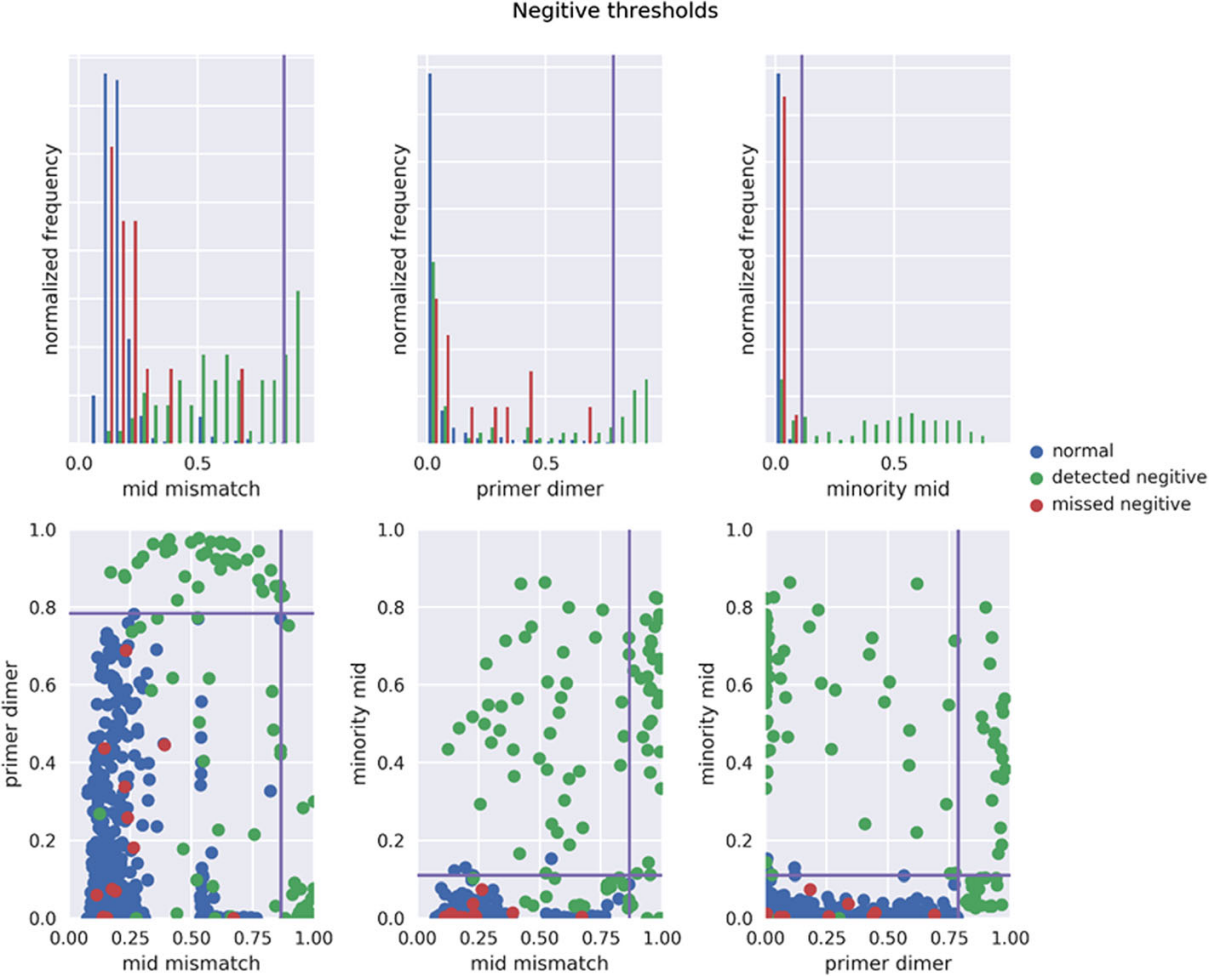
Breakdown of data categorizations using parameters from the three filter threshold combination. Top row shows histograms of each of the three filters. Bottom row shows results of using any two of the filters alone

At the minimal Gini impurity index of 0.0089, the resultant thresholds were 0.785, 0.865, 0.155 for the primer dimer, MID mismatch, and minority MID filters, respectively, with an accuracy of 0.9910, f1-score of 0.9289, an excluded proportion of 0.0516, TP = 98 (0.9899) and TN = 1554 (0.9911). This threshold combination identifies 5.9% of the combined PNN, PN, and FN as putative negatives or samples with loss of product (Fig. 7). Application of the 3-threshold combination to the FNN set categorizes 69.0% (290/420) as negative or loss of product. Applying any 2 of the 3 filters used in the 3-threshold combination resulted in missed negative calls in all cases; however, when applying only the primer dimer and minority MID filters, the missed negative calls were minimal (Fig. 8). Application of these two thresholds only to the FNN resulted in 66.4% categorized as a negative or loss of product (279/420). Calculation of the minimal Gini impurity index using only the primer dimer and minority MID filters yielded thresholds of 0.785 and 0.11 respectively with an accuracy of 0.9850, f1-score of 0.8804, an excluded proportion of 0.0330, TP = 92 (0.94850) and TN = 1550 (0.98730).

### Genotyping

**Fig. 9:**
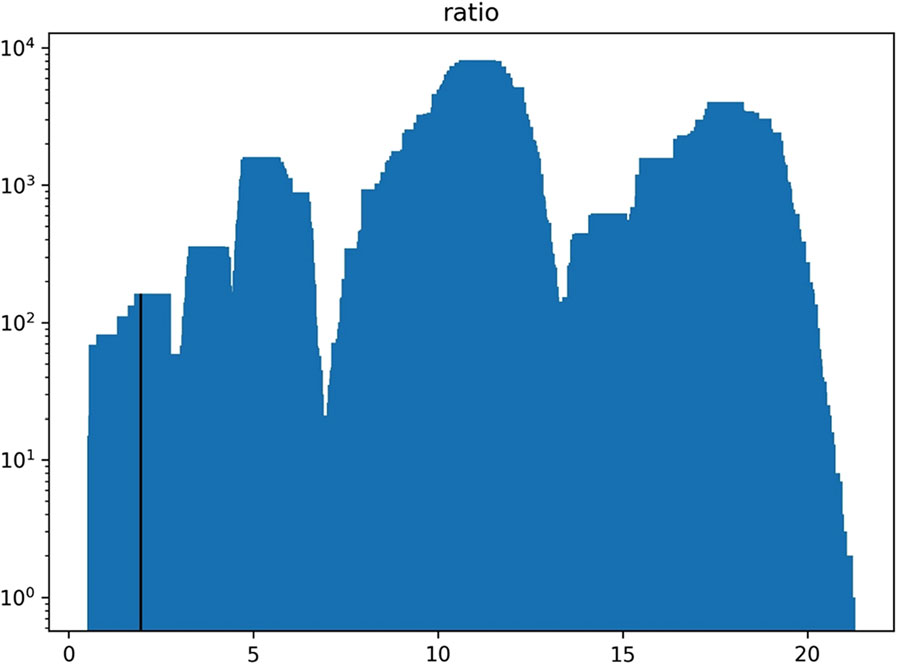
Histogram of the ratio of bit score-derived log probabilities of best to second-best subtype matches of the sequences in all deduplicated samples submitted to GHOST. Solid line indicates the cutoff ratio of 2, with the area under the curve to the left of the cutoff representing unique sequences that are classified only at the genotype level

For subtype classification, out of the 11,538,371 unique sequences evaluated from the 2087 samples, 323,862 (2.81%) had no subtype match, majority (*n* = 10,093,688; 87.58%) had only one subtype match, 1,111,483 (9.63%) had 2 subtype matches, and 9338 had 3 subtype matches. None had 4 or more subtype matches. For those with ≥2 matches, a majority of log probability ratios of the best match divided by the second-best match were found to have values between 8 and 18 (Fig. 9). 1,118,938 (9.70%) sequences had a corresponding ratio less than 2, of which 1,118,754 (99.98% of those with ratio < 2) exclusively involved subtypes of genotype 1, 179 exclusively involved subtypes of genotype 2, and 5 involved hits between 3a and either 1a or 1b. (Table 4).

**Table 4:** Sequence counts for unique sequences found to have a ratio of first to second-best hit scores under 2

major	minor	count	proportion
1a	1c	704,704	0.057086
1b	1c	407,112	0.032979
1a	1b	3548	0.000287
1b	1a	3390	0.000275
2a	2c	172	1.39E-05
2c	2a	7	5.67E-07
3a	1a	3	2.43E-07
1b	3a	2	1.62E-07

Sequences unable to be genotyped were tallied from all samples submitted to GHOST; 828 of the 2087 (39.7%) samples had a total frequency of > 0. For 65 samples, unmatched was the dominant genotype category, with dominance for a genotype category within a sample being defined as having the highest total frequency of sequences.

**Fig. 10:**
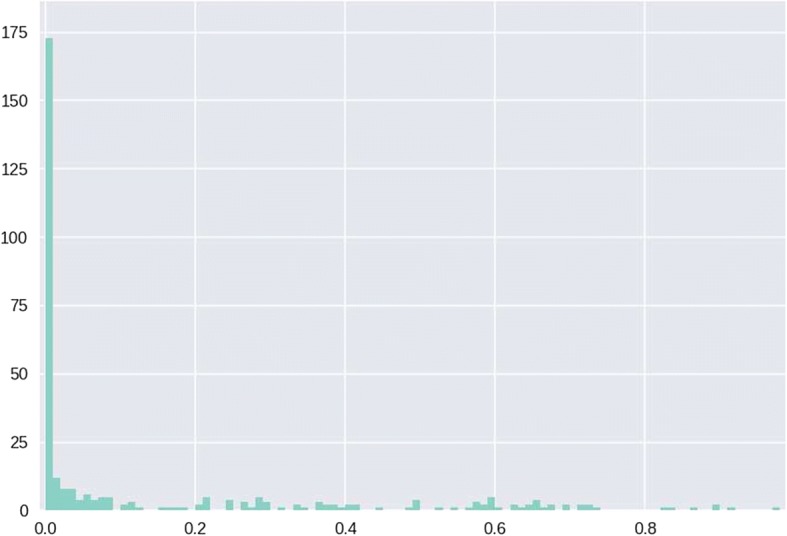
Histogram of prevalence ratios for all non-dominant subtypes where prevalence ratio is defined as the total frequency of the subtype divided by the total frequency of the dominant type

Of all 2320 subtype classifications made in all samples, 406 (17.5%) were non-dominant, and 1914 (82.5%) were dominant. The relative prevalence (prevalence ratio) of the subtype was calculated by the ratio of total frequency of the subtype divided by the total frequency of the dominant subtype (Fig. 10). The prevalence ratios had a mean of 0.113264 and a median of 0.008815.

### Influences on depth

Multiplexing level and flowcell type are the primary factors in defining sequencing depth. All libraries used for sequencing were paired-end using the Illumina MiSeq Reagent Kits v2 (500 cycles), v2 nano (500 cycles), and v3 (600 cycles) with the multiplexing level ranging from 8 to 96 samples per run. Applying logistic regression to either kit type or multiplex level to determine the effect on QC task passage yielded no statistical support towards a correlation.

### Linkage network of all samples

**Fig. 11:**
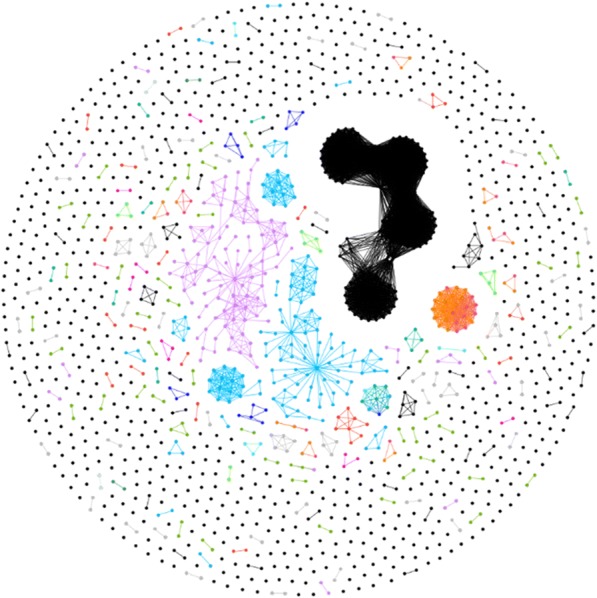
All deduplicated samples submitted to GHOST, including artificially created panel verification samples and non-linking samples. Node and link colors were arbitrarily assigned to clusters

**Fig. 12:**
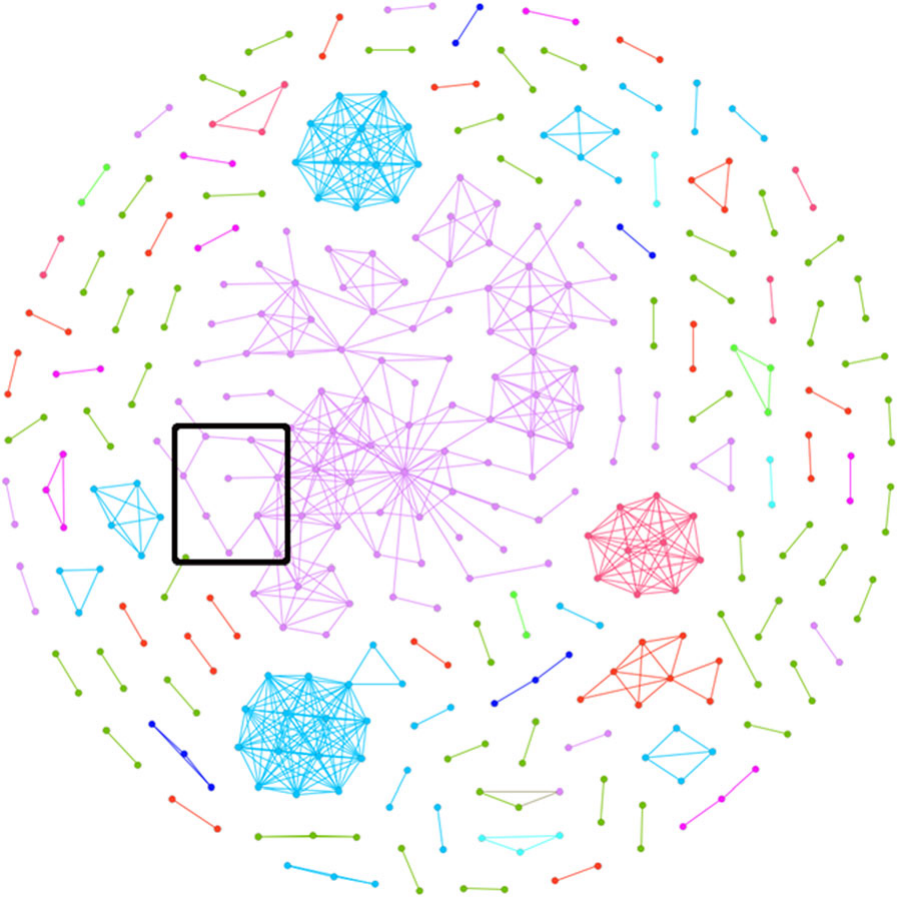
All links found in GHOST. Nodes representing samples artificially created for panel verifications by state pilot participants were removed, along with non-linking samples. Box encloses a chordless cycle. Node and link colors were arbitrarily assigned to clusters

**Fig. 13:**
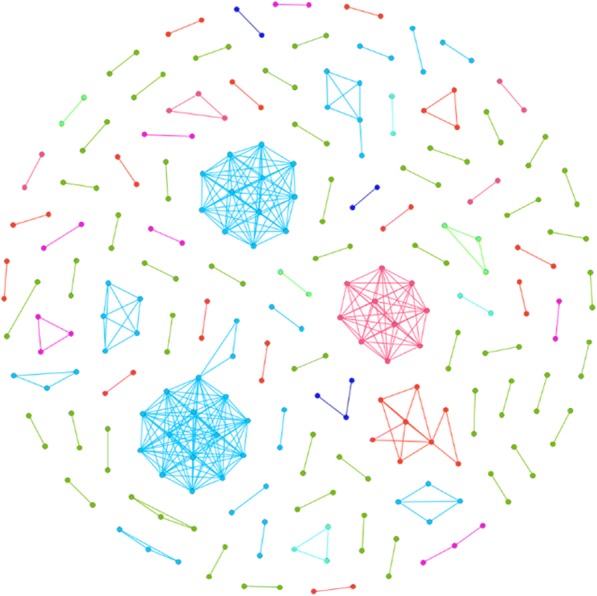
All links found in GHOST with removal of nodes representing samples artificially created for panel verifications by state pilot participants and nodes representing samples associated with a project with known quality control issues. Non-linking samples removed. Node and link colors were arbitrarily assigned to clusters

All samples passing QC tasks to-date were evaluated together in linkage analysis to check for cross-site linkage or any other type of anomaly (Fig. 11). Non-linking nodes were removed for clarity as well as all samples that originate from libraries created artificially using stock sera (Fig. 12). The network was visually dominated by a large irregular and asymmetric cluster. It was found that this cluster represented a project with multiple quality impairing issues including sample collection, handling, and known mistakes in library preparation. A type of particularly unusual feature was observed in this cluster – a set of nodes comprising a closed linkage cycle such that all edges between nodes in the cycle are a part of the cycle, and no other edges exist between nodes in the cycle that are not part of the cycle itself. This type of cycle is called a “chordless cycle”. Owing to the aforementioned problems, the sample set containing these cycles was removed to observe the linkage remaining (Fig. 13).

## Discussion

### Beyond a molecular surveillance system

The properties of the GHOST system allow one to detect and monitor HCV outbreaks and guide intervention using molecular techniques. Therefore, this system can be categorized as a variation of a cyber-molecular surveillance system for the detection of HCV transmission events. With many systems, there is an implied independent relationship between the validity of the testing techniques and the quality of the input and corresponding output, which is typically the responsibility of the investigator performing the test. This partition of responsibility gives rise to the so-called Garbage-In-Garbage-Out model and presents an unacceptable scenario for the GHOST system because it allows for the potential introduction of false results, misspent effort, and faulty downstream analyses and surveillance activities. It is for these reasons that we have endeavored to add multiple layers of automatic quality control to the GHOST system. These layers include: i) automatic curation, ii) identification and elimination of erroneous data, iii) error diagnostics, iv) automatic reporting of abnormalities, v) identification of the error causes, and vi) suggested course of action for abnormality mitigation.

### Filter observations

Elevations in read pairs discarded by the Casper mismatch and primer dimer filters reflect two very common issues affecting samples or entire runs – poor PHRED qualities and inadequate purification or loss of product in the library preparation, respectively. Both issues exist on a spectrum that permeate a significant proportion of data submitted and should not warrant rejection when levels are in moderation because the GHOST system is adept in filtering the affected data. However, it would be beneficial to warn the user when either of these filter percentages rise to a moderate level, so that the laboratorian may take corrective action in subsequent testing.

Therefore, a warning is displayed for samples with primer dimer filter percentages that have surpassed 0.3681 demarcating 95% of PNN data in the percentage of read pairs discarded (Table 5). Fig. 4 shows that as percentage surpasses approximately 80%, other filters also begin to show elevations in the percentage discarded, indicating a problem that may transcend purification issues. It was also observed that the primer dimer, MID mismatch, and minority MID filters all showed a significant difference in means in the comparison of PN and PNN. We found that primer dimer and minority MID filters alone with thresholds of 0.785 and 0.11, respectively, performed nearly as well in discrimination of negative samples from passing samples with only a 0.006 difference in accuracy from the 3 thresholds derived from all three filters. Given that there is a known occurrence of some negative controls that were either mislabeled or contaminated, it’s not known which set of thresholds actually performs better at discriminating true negatives or losses of product from standard samples. Furthermore, it can be reasoned that because the Illumina sequencing technology is such a powerful tool with respect to sequencing depth and sensitivity, in the absence of product one might see either an amplification of trace cross-contamination indicated by the elevated rates in the minority MID filter, or an amplification of trace amounts of short products not intended to be amplified, indicated by elevated rates in primer-dimer filter, or both. The role of the MID mismatch filter is not immediately clear. Therefore, the 2-threshold test using the primer dimer and minority MID filters is employed to label a sample as either a negative or product loss and exclude it from analysis results. The user is notified of the sample classification (Table 5).

**Table 5:** Quality Control event descriptions, triggers, actions, and notifications

Column1	Event	Action	Indicator	Notification	Suggestion
A	Poor quality	Warn	Casper mismatch filter > 95% PNN data	In sample X, the Casper alignment step discarded high level of pairs due to mismatches.	Please ensure the quality of amplification reagents not compromised (check polymerase expiration date, proper storage conditions, quality of primers). Please confirm the concentration and quality of pooled library and ensure the correct concentration loaded on the chip. Check the expiration date on the MiSeq reagent kit.
B	Poor purification	Warn	Primer dimer filter > 95% PNN data	In sample X, the primer dimer filter shows a high level of pairs discarded	Please check the concentration of primers (barcode and index) and review magnetic beads cleaning procedure. Ensure the quality of your final pooled library exceeds 95% purity.
C	Negative or loss of product detection	Reject, warn	Primer dimer filter > 0.785 or minority MID filter > 0.11	Sample X was determined to be either a negative control or suffered a loss of product during library preparation.	If this was not intended to be a negative control, please check samples proximity to an intended negative control, and if the negative control passes, consider that there may have been mislaballing. Please repeat the library preparation for this sample.
D	Unclassified sequences form dominant population for sample	Warn, notify CDC	Proportion of sequences that cannot be classified is higher than for any other subtype.	Sample X cannot be classified. CDC/DVH has been notified.	Please wait to be contacted by CDC staff.
E	Subtype classification issues	Warn	Sequences within the population has a best subtype match and second best subtype match with ratio < 2	For sample X, ambiguous subtype classifications have been detected.	Please note that this sample’s subtype is questionable.
F	Chordless cycle detected	Warn, notify CDC	Analysis task contains a chordless cycle of 4 nodes or more	Chordless cycle detected in samples X, Y, and Z.	Please check for signs of contamination between samples X, Y, and Z, and repeat library if feasible.
G	Residual read-pair level too low	Reject	Read pair count < 10,000 after all other filters execute	Sample X does not have enough reads after all filters execute to proceed.	Please review the sample preparation for sample X. Repeat this sample in next library if feasible. If not, contact ghost@cdc.gov about relaxing read pair level restrictions.

Lastly, we observed that in some cases, after random sampling, read pairs were significantly reduced due to excessive read removal by either a particular filter or combination of filters for reasons that are not readily apparent. We previously determined that that linkage results can be reliably recovered when subsampling at a level of 10,000 read pairs [12]. We introduce a final check that rejects samples containing < 10,000 residual reads after all other filters have completed (Table 5). This filter would reject 17.9% (313/1750) of the PNN samples and 27.3% (3/11) of the PN samples.

### Chordless cycles

In the contaminated linked cluster referenced in Fig. 11, multiple subsets of the cluster contain a chordless cycle. This type of occurrence would not likely be a naturally occurring transmission event except in the cases of i) multiple infections, ii) a multitude of unsampled individuals comprising a much more complex underlying network, or iii) convergent evolution. The detection of convergence is, however, highly implausible for the sample size analyzed in this study.

Given that it is known that there were issues in the collection, storage, and handling of the material sources for these samples, and a known contamination event in library preparation using these material sources, we believe these instances to be an artifact of false linkage caused by laboratory contamination. To flag future similar events, an automatic check for chordless cycles is conducted within GHOST graphical outputs, and those found are reported as an anomaly to be further investigated (Table 5).

### Genotyping caveats

Sequences in samples that cannot be classified as a subtype could be an indication of multiple issues. One scenario would be the emergence of a new strain that is not in our reference database. In high risk populations, it is possible that a chimeric strain could arise signaling unusual transmission rates or patterns. Other causes could be technical in nature, owing to the cleaning and linking algorithms, or to an unusual event in the library preparation. In each of these cases, we would want to be aware of this occurrence, and we have classified the existence of sequences of an undetermined subtype as an anomaly to be recorded in the QC task report (Table 5).

Because of the uncertainty of distinguishing some HCV strains that belong to different subtypes of the same genotype using the HCV genomic region encoding E1/E2 junction applied in GHOST [12, 13], the genotyper was adjusted to only classify at the genotype level if the ratio of the log probabilities between the best and second-best log matches is less than 2. This threshold is somewhat arbitrary, however, we feel it provides a moderate level of confidence at the price of a relatively modest level of exclusivity as illustrated in Fig. 9.

Non-dominant subtypes with low frequencies relative to the dominant subtype were found to be common, and likely arise mostly from minor contamination. A filter in the Analysis task already exists that restricts linkage between sequences of samples when the maximum frequency is below a threshold (currently 10).

### Future directions

The utmost data quality is difficult to achieve because of unclear criteria for such a task. In GHOST, the major criterion for QC is accuracy of public health information generated by the system. In its current version, the GHOST QC module controls data errors affecting the identification of transmission links among HCV strains. However, addition of new analytical models for other pathogens or for the detection of other parameters of HCV infection important for public health, such as recent infection, sensitivity to drugs, disease severity, etc., may significantly change QC requirements and opens new directions for research. With the continuing application of GHOST in different epidemiological settings by many users, we expect accumulation of sufficient data to improve further QC using automatically updatable models specifically calibrated for each MiSeq run and user.

## Conclusions

GHOST, a novel technology, can also face unique challenges as it is being routinely used. Initially, many of these challenges can be difficult to approach due to many unknown factors of HCV infection and multitude of potential experimental artefacts; however, piloting of the technology enabled the identification of several new QC problems affecting the GHOST performance and accuracy. New QC models developed here improved protection of the system and users from erroneous data and inaccurate inferences. GHOST was upgraded to include new functions for the identification of causes of erroneous data and recommendation of corrective actions to laboratory users to facilitate evolution of the entire system towards becoming an autonomous expert system for guiding public health interventions.

## Abbreviations

CDC: Centers for Disease Control and Prevention
EPLD: End-Point Limiting-Dilution
FN: Failing Negative
FNN: Failing Non-Negative
GHOST: Global Hepatitis Outbreak and Surveillance Technology
HCV: Hepatitis C Virus
NGS: Next Generation Sequencing
PII: Personally Identifiable Information
PN: Passing Negative
PNN: Passing Non-Negative
QC: Quality Control
